# The sociocultural and behavioural characteristics that patients want in psychiatrists: cross-sectional survey of patients’ views

**DOI:** 10.1192/bjb.2020.115

**Published:** 2021-06

**Authors:** Richard Laugharne, Stefan Priebe, Agnes Chevalier, Catherine Paton, Rajaei K. Sharma, Allison O'Kelly, Giles Richards

**Affiliations:** 1 Cornwall Partnership NHS Foundation Trust, Liskeard, UK; 2 Unit for Social and Community Psychiatry, Queen Mary University of London, UK; 3 Devon Partnership NHS Trust, Newton Abbot, UK; 4 University of Exeter Medical School, UK

**Keywords:** Patient involvement, community mental health teams, gender, psychiatrists, preference

## Abstract

**Aims and method:**

There appears to be no research to date investigating patients’ preferences for sociocultural characteristics or behavioural qualities of psychiatrists. We aimed to assess which are most important to patients. Patients (132) in community mental health teams across two sites (East Cornwall, East London) completed a questionnaire ranking the importance of different sociocultural characteristics and behaviours of psychiatrists.

**Results:**

Patients cared more about age and gender than other characteristics. Four preferences (from a choice of ten) regarding behavioural qualities were clearly identified as important: explaining things clearly, dedication to personal treatment, being friendly and polite, and being up to date with medical knowledge.

**Clinical implications:**

Patients are fairly unconcerned about the age, gender, religion and social background of psychiatrists. Characteristics they care about most include communication skills, competence, dedication to personal treatment and friendliness. Explaining things clearly is particularly important. This indicates specific areas of improvement for training and further research.

Patient preference is a central principle in healthcare. Both patient views and satisfaction are recognised as important as expectations of standards of care rise.^1^ Studies on patient satisfaction with care have shown that the therapeutic relationship between patient and doctor and the interpersonal relationship with staff are important to patients.^2^

The evolution of patient-centred care means that patient involvement is increasingly integral to health services research and development, demonstrated by a rapidly growing literature base of patient views. However, there is still a dearth of literature examining patient involvement for improving professional performance in medicine.^3^ The literature that does exist largely focuses on communication skills during consultations. This scarcity means that we cannot yet state whether patient feedback can affect performance and what the influential factors are.^4^

We know that judgements are made on the basis of initial perceptions;^5–7^ these perceptions are based on easily identifiable features such as gender or age, and on traits judged to be important by each individual, such as standing within society. Gledhill et al found that psychiatric in-patients prefer psychiatrists to wear smart attire and to call them by their first name, although this research was conducted in 1997.^8^ However, the smart attire may also lead to patients viewing their psychiatrist as less friendly and approachable.

Patient preference regarding a doctor's gender is an obvious and better explored example of consideration of patients’ attitudes. It has been found that significant gender preference is low but trends for same-sex doctors are seen in specific scenarios, including choosing a primary care doctor.^9^

A study undertaken in The Netherlands in 1993 by Kerssens et al used a general household survey to investigate gender preference for 13 different medical specialties and explored possible reasons for any preferences arising. They found that gender preference was stronger in specialties more likely to be engaged in intimate and psychosocial health problems, such as general practitioners (GPs) and gynaecologists. They found that individuals who indicated a preference for a female physician did so on the basis that they found it easier to talk to a female and felt more comfortable being examined by a female and the same reasons were cited by those indicating a preference for a male doctor. For women, 81% had no gender preference for psychiatrists, 4% preferred a male and 15% a female psychiatrist. For men, 91% had no preference, with 3% preferring a female and 6% a male psychiatrist. This was a population, not a patient, survey.^10^

More recently it has been suggested that gender is likely to continue to influence the doctor–patient relationship more in psychiatry than in most other specialties. This may be due to the many entrenched social perceptions and stereotypes that we are still too unaware of.^11^ It has also been found that female psychiatrists are still at an advantage when it comes to developing a working relationship with their patients.^12^

Patients are also likely to have strong views on how important various behaviours and skills of clinicians are. When examining communication, there is clear evidence that modifiable human behaviours can have positive or negative effects in consultations. Yet even when specifically examining empathy, Derksen et al found that, although widely promoted as a fundamental skill in clinical practice, evidence is scarce for the effect of greater empathy.^13^ A commonly identified negative characteristic of healthcare professionals is paternalism. The desire for an equal power dynamic is one theme that frequently arises in studies examining the patient–medical professional relationship.^14^

Evidence suggests that patients attending out-patient psychiatric services are generally satisfied with the care they receive from their psychiatrists.^1,12^ There is some evidence exploring patient satisfaction pertaining to particular qualities in their psychiatrists, such as whether they are attentive, caring in demeanour, knowledgeable about an individual's illness and able to explain conditions well.^15–17^

There is little literature on any aspect of how the patients’ role is integrated.^4^ Even when patient involvement is promoted, many assumptions are made as to the scope, such as how, when and on what they can give feedback. Indeed, it has been seen that there is sometimes a misalignment between patient priorities and changes put into effect.^18^ For example, as part of the revalidation cycle in the UK's National Health Service (NHS), doctors are mandated to submit and evaluate patient feedback. This has been found to have a positive influence overall although its exact purpose and use remain a point of contention for many.^3^

It is also important to question why patient involvement in the development of professional performance has been lacking. Recent analyses have found that negative attitudes of doctors may in fact be a key barrier preventing systems development, thus hindering performance improvement.^3^ It is still important to generate the evidence, as clinical outcomes are likely to be affected.

There is also some indication that a therapist's perception of the patient's priorities can be incorrect. When there is a developing relationship, this failure can strongly affect the patient's confidence in their therapist.^19^ However, there appears to be no research to date specifically investigating patients’ preferences for the sociocultural characteristics of their psychiatrists.

It can take up to 17 years for research to translate into practice in the UK health service; by developing and improving patient involvement we may be able to improve this implementation process and decrease the time frame.^20^

## Aims

This study aimed to explore the characteristics and qualities of psychiatrists that are most important to patients. We asked the following research questions:

- What sociocultural characteristics about psychiatrists are important to patients?
- What behaviours are most important to patients in their psychiatrist?

In addition, we hoped the data would be able to shed light on the following gender-based question:

- Are female patients more likely to want a female psychiatrist?

## Method

### Setting

The study took place in community mental health teams (CMHTs) across two UK NHS foundation trusts. The sites were a general CMHT and a complex care and dementia team in East Cornwall and a CMHT in East London. We therefore approached patients across very different environments – a deprived rural area in south-east Cornwall, which is predominantly White in ethnicity, and a deprived urban area in London, which is significantly ethnically diverse.

### Design

This was an exploratory cross-sectional survey of patients’ views.

### Participants

Patients were identified from the team case-loads. They were included if they were over the age of 18 years, had contact with a psychiatrist within secondary mental health services and were classified as having a severe and enduring mental illness, which included patients with a psychotic illness (for example schizophrenia or bipolar affective disorder), a severe depressive disorder, a personality disorder or dementia. Patients were excluded if they were acutely unwell and therefore lacked capacity to give consent and if they were unable to speak English.

### Data collection

In East Cornwall, patients were initially approached via their care coordinator during a pre-existing appointment or following an appointment with their psychiatrist. This initial approach resulted in a fairly low response rate, so an amendment to the study's ethical approval was sought and patients were also approached by a mail shot. In East London, patients were approached via a mail shot after they had been identified by a researcher in conjunction with their care coordinator.

Participants completed a brief questionnaire which asked them about several non-modifiable sociocultural characteristics of psychiatrists, including age, gender, religion, social background and marital status. They were asked to state whether or not they had a preference with regard to the gender, age or level of experience of their psychiatrist. Then the participant was asked to state how important each characteristic was. Finally, they were asked about modifiable characteristics. The participant was asked to select and rank the three qualities/behaviours most important to them from a list of ten:

- the psychiatrist is friendly and polite in manner
- the psychiatrist is recommended as good by other patients
- the psychiatrist is recommended as good by my GP
- the psychiatrist is actively involved in scientific research
- the psychiatrist is up to date with medical knowledge
- the psychiatrist has a professional appearance and is well dressed
- the psychiatrist is dedicated to my personal treatment
- the psychiatrist is positive and optimistic
- the psychiatrist explains things to me
- the psychiatrist has a similar social and cultural background to me.

This list of behaviours was generated from discussions within the research team and consideration of the literature.^1,13,15^

Researchers then collected sociodemographic details about the patients from computerised medical records, including their age and gender.

All data collected were strictly anonymised to prevent patient identification.

### Data analysis

The overall results were compiled to reveal:

- preference for gender
- preference for age
- preference for experience
- importance of the sociocultural characteristics
- ranking in importance for the ten characteristics.

Comparison was then made to see whether female patients had a preference for seeing a female psychiatrist.

### Ethical approval

The study received research ethical approval (REC reference number 13/EE/0230) from the National Research Ethics Committee East of England.

### Gratuity

Participants were offered £5 (cash in East Cornwall and a voucher in East London) as a token of appreciation for their time. This was not advertised in the patient information leaflet, to reduce potential response bias.

## Results

We received 132 returns of the questionnaire across all sites (76 from the East Cornwall CMHT, 28 from the East Cornwall complex care and dementia team and 28 from the East London CMHT). Participants were aged over 18 years, treated in secondary mental healthcare and were diagnosed with a severe and enduring mental illness.

### The sociocultural characteristics important to patients

**Fig. 1:**
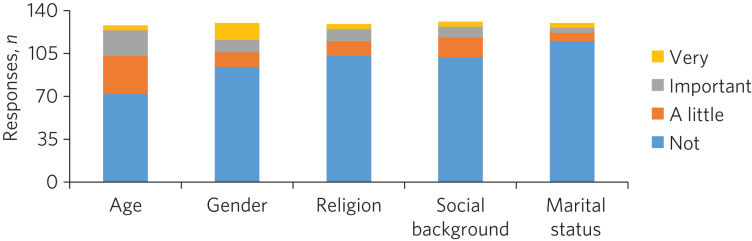
Participants’ rating of the importance of their psychiatrist's sociocultural characteristics.

Participants cared more about the age and gender of their psychiatrist than their religion, background and marital status, but the majority of participants were not concerned about any of these factors (Fig. 1). With regard to age, 28% of the total sample expressed a preference regarding the age of their psychiatrist: 16% preferred a psychiatrist under 40 years old, 73% a psychiatrist 40–55 years and 11% a psychiatrist over 55 years. A larger proportion of the total sample (61%) expressed a preference regarding the level of experience of their psychiatrist, with 79% of them stating a preference for a psychiatrist who had been qualified for some time.

### Behaviours most important to patients

**Fig. 2:**
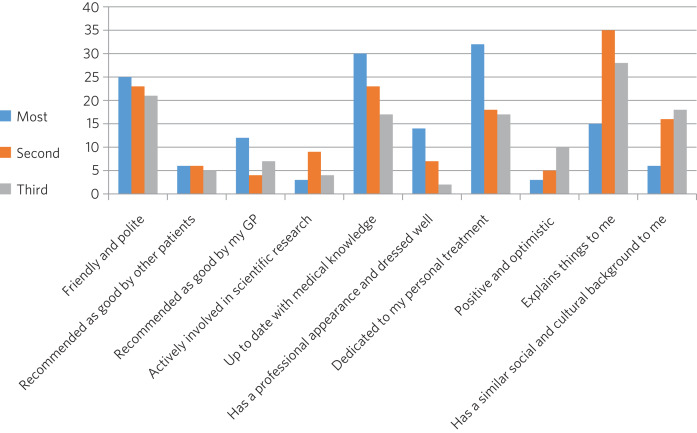
Participants’ ranking of the top three (out of ten) preferred qualities/behaviours shown by their psychiatrist.

When asked to rank the three most important qualities/behaviours from the list of ten, there were four clear preferences (Fig. 2):

- the psychiatrist explains things to me (more than two-thirds had this in their top three rankings)
- the psychiatrist is dedicated to my personal treatment
- the psychiatrist is up to date with medical knowledge
- the psychiatrist is friendly and polite.

### Additional results regarding gender preference

**Fig. 3:**
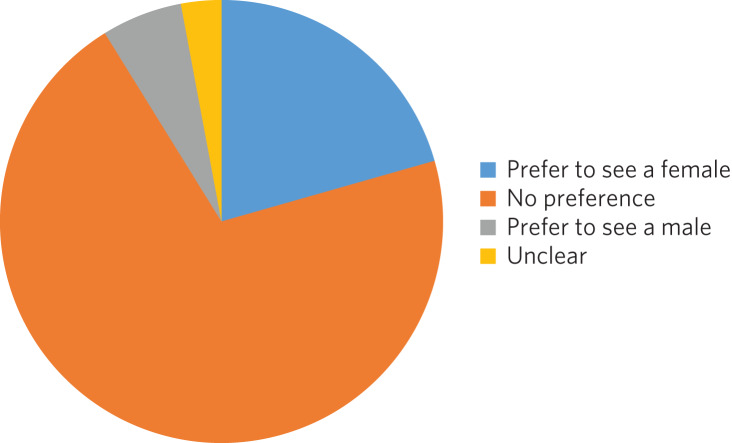
Female participants’ preference for the gender of their psychiatrist.

In total 73 women completed the questionnaire; 73% expressed no preference regarding the gender of their psychiatrist (Fig. 3). A similar percentage was observed among the 59 men who completed the questionnaire: 75% expressed no preference with regard to the gender of their psychiatrist. There was no significant difference between genders at the 5% level on statistical analysis (chi-squared test of independence, 5% confidence value).

## Discussion

### Main findings

In this study the characteristics of psychiatrists that patients cared most about included communication skills, competence, dedication to personal treatment and friendliness. Being able to explain things to patients was particularly important. Of note, being recommended by GPs and other patients was not as important, nor was appearance or being positive and optimistic. The importance of ‘dedication to personal treatment’ supports early findings by Johansson & Eklund that a common priority of psychiatric patients is the development of a therapeutic relationship.^19^

Participants did not express strong preferences about the age, gender, religion, social background or marital status of their psychiatrist.

As regards the modifiable characteristics analysed, participants did not identify optimism as being important. This aspect of the therapeutic relationship is a quality assessed in some consultant 360-degree appraisal systems. Our finding may be due to a desire for the clinician to be realistic and a feeling that being unduly optimistic can give false hope. As the survey population was patients in secondary care, there may be contributing factors that were not taken into account. These might include the chronicity of specific conditions and the amount of time that the participants have been receiving care.

Another postulation is that the questionnaire asked about a psychiatrist being positive and optimistic; patients might construe a combination of positivity and optimism as lacking in empathy and not understanding their suffering or recognising the impact their presentation/illness is having on their life.

In terms of non-modifiable characteristics, none were found to have significant importance. The preference for age and experience was of note, as it suggests that more senior clinicians have characteristics desired by patients.

With gender preference, the female participants did not show an overall preference to see a female psychiatrist. This is a comparable finding to the population survey undertaken in The Netherlands in which the majority of both women and men expressed no preference about the gender of the psychiatrist seen.^10^ The conflict with more recent studies into gender bias among psychiatric patients may be due to the disparity between preconceptions and outcomes with male/female psychiatrists. This warrants a focused analysis that could be instrumental to professional improvement.

### Limitations

We must consider the potential limitations of the study, in particular response bias. One of the factors specifically commented on by the researcher based in East London was the fact that patients were more likely to return a questionnaire if they had previously met her in an earlier role running therapeutic groups in a hospital setting. In conjunction with patient-experience surveys generally having low response rates, this bias may be notable.^21^

The study was also limited to people who spoke English: although this may not have had a significant impact on the results in the East Cornwall sites (nobody on the East Cornwall CMHT case-load required the use of an interpreter or did not speak English as a first or second language at the time of the study), there is a considerably more culturally diverse population in East London who could not then be approached.

In terms of study design, there is no validated questionnaire specific enough to the aims of this survey and applicable to the setting. The behavioural qualities listed in the study were determined through discussion among clinician-researchers. The list might have been strengthened with input from patients.

We did not use a mixed-methods approach owing to limited study resources. Analysing the data by patient characteristics, including experience of services and diagnosed disorder, would have given more insight from a patient perspective, and may be an opportunity for future research.

### Implications

Although we may worry about a patient's perception of us based on physical, usually unchangeable characteristics, our focus should be on how we communicate with our patients, as this appears to have more importance for patients. We should not underestimate the significance of being friendly in our clinical work, but also remember that patients value the time-honoured importance of up-to-date knowledge and being dedicated to their personal care.

This research focused on patients in secondary care, many of whom are already experienced with regard to psychiatric treatment. With this in mind, consideration should be given to repeating the research with newly referred patients.

It should also be considered that, in circumstances where the relationship between a patient and their psychiatrist has broken down and a new psychiatrist is to be allocated, attention to matching the psychiatrist and patient on the basis of sociodemographic characteristics is not merited by the evidence.

Some of the behaviours that were identified as important can be trained and regulating authorities such as the General Medical Council and the Care Quality Commission may wish to consider greater encouragement in developing these skills. Psychiatrists are already expected to update their knowledge through continuing professional development, but there is limited systematic training or supervision on how psychiatrists should explain treatments to patients. These communication skills are important to patients.

## Data Availability

Data is available from the corresponding author.

## About the authors

**Richard Laugharne**, FRCPsych, is a consultant psychiatrist with Cornwall Partnership NHS Foundation Trust, working in Trevillis House, Liskeard, UK. **Stefan Priebe**, FRCPsych, is Professor of Community Psychiatry in the Unit for Social and Community Psychiatry, a World Health Organization Collaborating Centre for Mental Health Service Development at Queen Mary University of London, UK. **Agnes Chevalier**, MSc, is a researcher in the Unit for Social and Community Psychiatry, Queen Mary University of London, UK. **Catherine Paton**, MRCPsych, is a consultant psychiatrist with Devon Partnership NHS Trust, UK. **Rajaei K. Sharma**, BSc(Hons), PgCert, is a medical student at the University of Exeter Medical School, UK. **Alison O'Kelly**, MSc, is a nurse consultant with Cornwall Partnership NHS Foundation Trust, working in Trevillis House, Liskeard, UK. **Giles Richards**, FRCPsych, is a consultant psychiatrist with Cornwall Partnership NHS Foundation Trust, working in Trevillis House, Liskeard, UK.
